# Algorithms Research and Precision Comparison of Different Frequency Combinations of BDS-3\GPS\Galileo for Precise Point Positioning in Asia-Pacific Region

**DOI:** 10.3390/s23135935

**Published:** 2023-06-26

**Authors:** Meng Gao, Zhihua Cao, Ziheng Meng, Chunbo Tan, Huizhong Zhu, Lu Huang

**Affiliations:** 1 School of Geomatics, Liaoning Technical University (LNTU), Fuxin 123000, China; 472220813@stu.lntu.edu.cn (Z.C.); 472220847@stu.lntu.edu.cn (Z.M.); 472220856@stu.lntu.edu.cn (C.T.); zhuhuizhong@lntu.edu.cn (H.Z.); 2 The 54th Research Institute of China Electronics Technology Group Corporation, Shijiazhuang 050000, China; hlcetc54@163.com

**Keywords:** BDS-3, PPP, frequency combination, ionosphere-free

## Abstract

With the continuous construction and development of the BeiDou navigation satellite system (BDS), its positioning performance is constantly being improved. In this study, the positioning performance of different frequency combinations of BDS-3/GPS/Galileo in the Asia-Pacific region was investigated. The precision products of Wuhan University and the observation data of nine MGEX stations were selected to compare and analyze the B1I\B1C\B2a\B3I and L1\E1 pseudo-range Standard Point Positioning (SPP) and B1IB2a\B1IB3I\B1CB2a\B1CB3I\B2aB3I\L1L2\E1E5a precise point positioning (PPP) performance, while B1I\B3I\L1 SPP and B1IB3I PPP were investigated using BDS-2 with QZSS supplemented with BDS-3 and GPS. The experimental results showed that the positioning precision of BDS-3/GPS/Galileo SPP was in the order of B1C > E1 > L1 > B1I > B3I > B2a, and it was not significantly improved after BDS-2 and QZSS were added. Moreover, for the PPP of different frequency combinations, the convergence speed was in the order of L1L2 > B1IB3I > E1E5a > B1CB3I > B1CB2a > B1IB2a > B2aB3I. After adding BDS-2, B1IB3I improved by about 11% in static mode and 27% in kinematic mode, which was similar to the L1L2 frequency combination. The positioning precision of different frequency combinations of BDS-3/GPS/Galileo was B1IB3I > B1CB3I > L1L2 > E1E5a > B1B2a > B1CB2a > B2aB3I. In static mode, after adding BDS-2, B1IB3I did not show significant improvement in the plane direction, and showed ~61% improvement in the elevation direction, and ~67% in the three-dimensional (3D) direction. In kinematic mode, after adding BDS-2, B1IB3I was improved by about 16% in the E direction, the N direction did not show significant change, it improved by ~38% in the U direction and by ~70% in the 3D direction. In general, the positioning performance of BDS-3 was slightly better than those of GPS and Galileo in the Asia-Pacific region, and it is believed that with the continuous development of BDS, its positioning performance will surely be improved further.

## 1. Introduction

Global navigation satellite systems (GNSS) can provide high-quality positioning, navigation, and timing (PNT) services to users around the world. It is widely used in intelligent navigation, automatic driving, earthquake mitigation, urban management, transportation, and other industries, and it plays a very important role in national economic construction and military defense industry. The BeiDou Global Navigation Satellite System (BDS-3), the third step in the construction of China’s satellite navigation system, was completed in June 2020 and can now provide all-weather PNT services for global users [1,2]. Compared with BDS-2, BDS-3 satellite is composed of three Geostationary Earth Orbit (GEO) satellites, three Inclined Geo-synchronous Orbit (IGSO) satellites, and twenty-four Medium Earth Orbit (MEO) satellites. Based on the original B1I and B3I frequencies, B1C, B2a, and B2b frequencies are added and broadcast to global users [3,4]. At present, the ground station of multi-GNSS experiment (MGEX) is also gradually updating the receiver antenna that can accept the signal of BDS-3 new frequency point [5].

It is well known that in order to achieve centimeter- level or even millimeter-level positioning, Zumberge et al. proposed the theory of precise point positioning (PPP) [6]. Since then, with the continuous development of GNSS, many scholars proposed several new theories and methods around different function models, different system combinations, different frequency combinations, etc., and proved that the positioning precision of each satellite navigation system is constantly improving [7,8,9,10].

In recent years, domestic and foreign scholars devoted significant research efforts on the global service performance of BDS-3, by conducting various types of tests for a total of six types of services provided by BDS-3, including PNT, Satellite-based Augmentation, PPP, Regional Short Message Communication, Global Short Message Communication, and International Rescue [4,11,12], providing references for different types of BDS-3 users. Furthermore, significant research attempts were made on the BDS-3 real-time orbital product [13,14] and the PPP-B2b service performance [15], which prove that the performance of BDS-3 reached the international top level. Gu et al. analyzed the standard point positioning (SPP) and PPP precision of the BDS-2/3 B1I/B3I and B1C/B2a using the Wuhan University (WHU)-released observable-specific bias (OSB) products, and the results showed that the SPP performance did not change significantly with the addition of the OSB correction, while the PPP performance improved remarkably [16]. Moreover, some scholars studied the performance of PPP from the orbital clock products provided by IGS and the combination of multi-frequency PPP and multi-system PPP; however, the analysis focused more on the combination PPP of B1I and B3l frequencies, and less on the analysis of B1C and B2a frequencies PPP [17,18,19]. Zhang et al. (2020) compared the performance of PPP for each system and multi-system combination, and showed that BDS exhibited PPP performance, which was basically found to be consistent with other navigation satellite systems [20]. Zhang et al. (2021) evaluated B1C and B2a from carrier-to-noise ratio and coding quality, and compared them with B1I/B2I/B3I, GPS, Galileo, and QZSS, and the results showed that B1C and B2a exhibited better signal strength and higher precision [21]. Shi et al. (2020) showed that the BDS-3 signal quality was generally better than that of the BDS-2, and that the precision of SPP in the Asia-Pacific region was improved by 12% to 60% compared to the BDS-2 system [22]. Hu et al. (2022) evaluated the positioning performance of BDS after adding inter-system bias (ISB) parameters. In terms of PPP performance, BDS-3 is better than BDS-2 in positioning precision and convergence time, while BDS-2/3 showed better positioning precision [23]. Zhu et al. (2021) found that the signal quality, SPP, and PPP performance of BDS-3 B1C/B2a in the global region were comparable to those of GPS and Galileo [24]. Liu et al. (2022) researched the DCB correction method of BDS-3 new frequency and made a comparative analysis of positioning precision of different frequencies. The results showed that B1I frequency exhibited the best SPP precision in the Asia-Pacific region, B1C frequency was found to lead in other regions, after convergence, and the precision of BDS-3 B1C/B2a or B1I/B3I dual-frequency PPP was slightly lower than that of GPS L1/L2 [25]. Wang et al. (2021) evaluated the B1C/B2a global regional dual-frequency PPP precision and compared it with GPS system, B1C/B2a was found to be worse than GPS L1L2 in terms of positioning precision in elevation direction, and comparable to GPS in terms of convergence [26]. Liu et al. (2021) compared BDS-3 B1C/B2a with BDS B1IB3I and found the convergence time of B1C/B2a similar to that of B1IB3I; nonetheless, its positioning precision was better than that of B1IB3I [27]. Shi et al. (2021) conducted single-frequency PPP experiments on B1I and B1C frequencies of BDS-2, BDS-3, and BDS-2/3, respectively, and the results showed that BDS-3 and BDS-2/3 PPP precision improved by about 60% compared to BDS-2 [28]. Cheng et al. (2022) [29] analyzed the positioning precision of BDS B1IB3I/B1CB2a and BDS-3 B1IB3I in polar regions. The results revealed that B1CB2a showed the highest positioning precision and the fastest convergence time in static mode, and BDS-3 B1IB3I exhibited the highest positioning precision and faster convergence in kinematic mode [29]. Li et al. (2020) proposed the BDS-3 optimal three-frequency signal linear combination, i.e., B1C/B1I/B2a, which could improve the positioning precision by about 20% compared with GPS L1L2 and Galileo E1E5a [30].

Currently, most researches on BDS-3 are based on quality analysis of its new frequency B1C/B2a signal and comparison of positioning precision of BDS in the global region. However, positioning performance of BDS-3 B1I/B1C/B2a/B3I frequency in the Asia-Pacific region was not extensively investigated; therefore, it is necessary to evaluate the positioning performance by combining each BDS-3 frequency. The method can provide an important data reference for other scholars’ research in this area, while it also can be quickly extended to any region, providing an effective validation solution for regional and global BDS-3 positioning performance analysis. Based on this, nine MGEX stations in the Asia-Pacific region were selected and precision products such as precision orbits and clock from the Analysis Center of WHU were used. Starting from the average number of visible satellites, position dilution of precision (PDOP), positioning precision, and convergence time, different frequency combinations of BDS-3, GPS, and Galileo were compared and analyzed, which proves that the positioning precision of BDS-3 is slightly better than those of GPS and Galileo in the Asia-Pacific region. Furthermore, based on the comparative analysis, optimal frequencies for SPP and optimal dual-frequency Ionosphere-free (IF) combinations for PPP of BDS-3 in Asia-Pacific region were picked out.

## 2. Mathematical Model

The observation equation for GNSS pseudo-range observations and carrier phase observations can be written as follows [31,32]:

(1) $$\begin{array}{c} P_{r,i}^{s,sys}=\rho_{r}^{s,sys}+c(dt_{r}-dt^{s,sys})+T_{r}^{s,sys}+\mu_{i}I_{r,1}^{s,sys}+d_{r,i}^{sys}-d_{i}^{s,sys}+e_{r,i}^{s,sys}\\ L_{r,i}^{s,B}=\rho_{r}^{s,sys}+c(dt_{r}-dt^{s,sys})+T_{r}^{s,sys}-\mu_{i}I_{r,1}^{s,sys}+\lambda_{i}^{sys}(b_{r,i}^{sys}-b_{i}^{s,sys})+\lambda_{i}^{B}N_{r,i}^{s,sys}+\varepsilon_{r,i}^{s,sys} \end{array}$$

where $P_{r,i}^{s,sys}$ is the pseudo-range observation; $L_{r,i}^{s,B}$ is the carrier phase observation; s is the satellite; r is the receiver; sys is the satellite system; i is the frequency number; $\rho_{r}^{s,sys}$ is the geometric distance from the satellite to the receiver; $dt_{r}$ and $dt^{s,sys}$ denote the receiver-satellite clock difference; and c is the speed of light. $T_{r}^{s,sys}$ is the tropospheric delay, the tropospheric wet delay is expressed as the product of the tropospheric wet delay in the zenith direction and the projection function, while the tropospheric dry delay is corrected by using the Saastamoinen model; $\mu_{i}$ is the ionospheric influence factor, and $\mu_{i}={\left(\frac{f_{1}^{sys}}{f_{i}^{sys}}\right)}^{2}$; $f_{i}^{sys}$ is the frequency value of a satellite system; $I_{r,1}^{s,sys}$ is the tilted path ionospheric delay for the first frequency; $d_{r,i}^{sys}$ and $d_{i}^{s,sys}$ denote the pseudo-range hardware delay between the receiver antenna and the signal processor and between the signal generation and the satellite antenna launch for a system frequency, respectively; $b_{r,i}^{sys}$ and $b_{i}^{s,sys}$ denote the phase hardware delay between the receiver antenna and the signal processor and between the signal generation and the satellite antenna launch for a system frequency; $\lambda_{i}$ is the i frequency wavelength; $N_{r,i}^{s}$ is the carrier phase circumference ambiguity; $e_{r,i}^{s,sys}$ and $\varepsilon_{r,i}^{s,sys}$ denote the observation noise and unmodeled error for the frequency pseudo-range and carrier phase observations.

IF is often used in PPP to eliminate the ionospheric first-order term, thus reducing the influence of ionospheric delay on GNSS observations, and its observation equation can be written as follows [33,34,35]:

(2) $$\begin{array}{c} P_{r,IF}^{s,sys}=\alpha_{12}P_{r,i}^{s,sys}+\beta_{12}P_{r,j}^{s,sys}=\rho_{r}^{s,sys}+(dt_{r}-dt^{s,sys})+T_{r}^{s,sys}+d_{r,IF}^{s,sys}+e_{r,IF}^{s,sys}\\ L_{r,IF}^{s,sys}=\alpha_{12}L_{r,i}^{s,sys}+\beta_{12}L_{r,j}^{s,sys}=\rho_{r}^{s,sys}+(dt_{r}-dt^{s,sys})+T_{r}^{s,sys}+\lambda_{IF}^{sys}(N_{r,IF}^{s,sys}+b_{r,IF}^{s,sys})+\varepsilon_{r,IF}^{s,sys} \end{array}$$

Among them,

(3) $$\left\{\begin{array}{l} d_{r,IF}^{s,sys}=d_{r,IF}^{sys}-d_{IF}^{s,sys}\\ b_{r,IF}^{s,sys}=b_{r,IF}^{sys}-b_{IF}^{s,sys} \end{array}\right.$$

(4) $$\left\{\begin{array}{l} d_{r,IF}^{sys}=\alpha_{12}d_{r,i}^{sys}+\beta_{12}d_{r,j}^{sys}\\ d_{IF}^{s,sys}=\alpha_{12}d_{i}^{s,sys}+\beta_{12}d_{j}^{s,sys}\\ b_{r,IF}^{sys}=\alpha_{12}b_{r,i}^{sys}+\beta_{12}b_{r,j}^{sys}\\ b_{IF}^{s,sys}=\alpha_{12}b_{i}^{s,sys}+\beta_{12}b_{j}^{s,sys} \end{array}\right.$$

(5) $$\left\{\begin{array}{l} \alpha_{12}=\frac{{\left(f_{i}^{sys}\right)}^{2}}{{\left(f_{i}^{sys}\right)}^{2}-{\left(f_{j}^{sys}\right)}^{2}}\\ \beta_{12}=-\frac{{\left(f_{j}^{sys}\right)}^{2}}{{\left(f_{i}^{sys}\right)}^{2}-{\left(f_{j}^{sys}\right)}^{2}} \end{array}\right.$$

where IF represents the term IF, $\alpha_{12}$ and $\beta_{12}$ are the correlation coefficients, i and j represent two different frequencies, the remaining symbols mean the same as those in Equation (1).

## 3. Experimental Analysis

**Figure 1:**
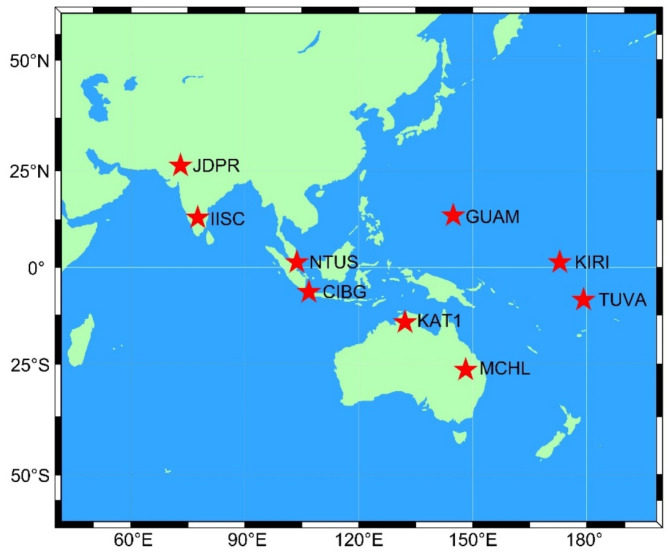
Distribution of stations.

To verify the positioning precision of BDS-3, GPS, and Galileo for SPP and PPP in the Asia-Pacific region, observations from nine MGEX stations from 6 to 12 February 2023 were selected for the experiment. The distribution of the station is shown in Figure 1, and the station name, latitude and longitude, station receiver type, antenna type, and frequency information are presented in Table 1.

The precision orbits and clock difference products used in the experiments were provided by the Analysis Center of WHU, and the SNX weekly solution file provided by IGS was used as the true value coordinates of the stations. The specific solution strategy is presented in Table 2.

**Figure 2:**
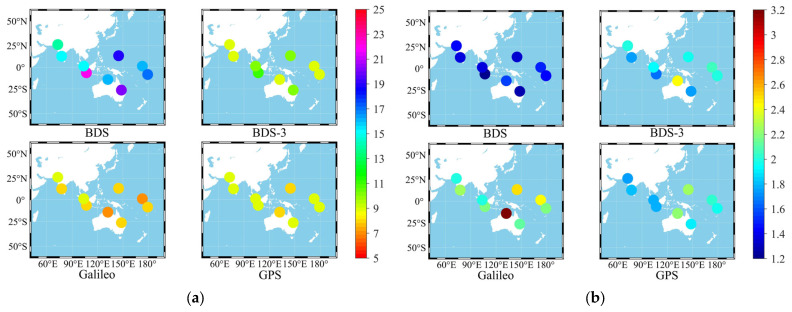
Average number of visible satellites and average PDOP of MGEX stations. (**a**) Average number of visible satellites, (**b**) Average satellites PDOP.

Figure 2 shows the average number of visible satellites and average PDOP of BDS, BDS-3, Galileo, and GPS that can be observed from the selected nine stations in a week.

Figure 2 demonstrates that BDS is better than BDS-3, Galileo, and GPS in terms of the number of satellites and PDOP; the number of satellites and PDOP of BDS-3 are slightly better than those of Galileo and GPS in some stations. This may be attributed to the different designs of BDS satellite constellation, operating altitude, orbital inclination, and other parameters. Consequently, the number of satellites and space configuration of BDS-3 in the Asia-Pacific region are better than those of other systems. Galileo is not much different from GPS in terms of the number of satellites; however, its PDOP is worse than those of the latter.

### 3.1. SPP Location Analysis

**Figure 3:**
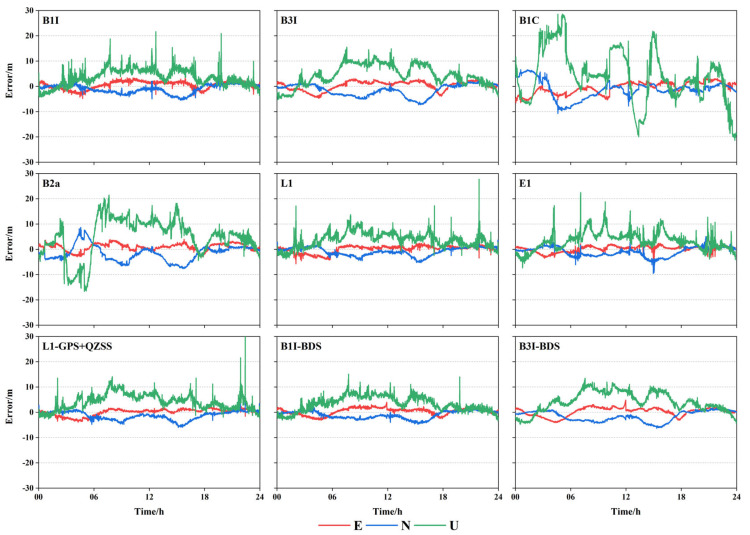
SPP time series of each frequency at IISC station.

In order to verify the SPP performance of BDS-3 in the Asia-Pacific region, nine stations in the Asia-Pacific region were selected and the data were processed for B1I/B3I/B1C/B2a/L1/E1 frequencies of BDS-3, Galileo, and GPS, respectively. To increase the experimental comparison, the B1I/B3I frequencies of BDS and L1 frequencies of GPS and QZSS combination were processed at the same time. Considering the IISC station as an example, the SPP time series graph of B1I/B3I/B1C/B2a/L1/E1/L1(GPS + QZSS)/B1I(BDS)/B3I(BDS) at DOY37 of the station was obtained, as shown in Figure 3.

Figure 3 illustrates that L1 and E1 exhibited the best positioning performance in the plane direction, B1I was the next best, while B3I, B1C, and B2a were slightly worse. However, after 12 h, the positioning precision of B1C improved and the fluctuation was small. After adding BDS-2, the positioning precision of B1I and B3I slightly improved, while that of L1 decreased after adding QZSS. In the elevation direction, the frequency precision was comparable except for B1C and B2a, and positioning precision of B1I, B3I, L1 was not significantly improved after adding BDS-2 and QZSS.

**Figure 4:**
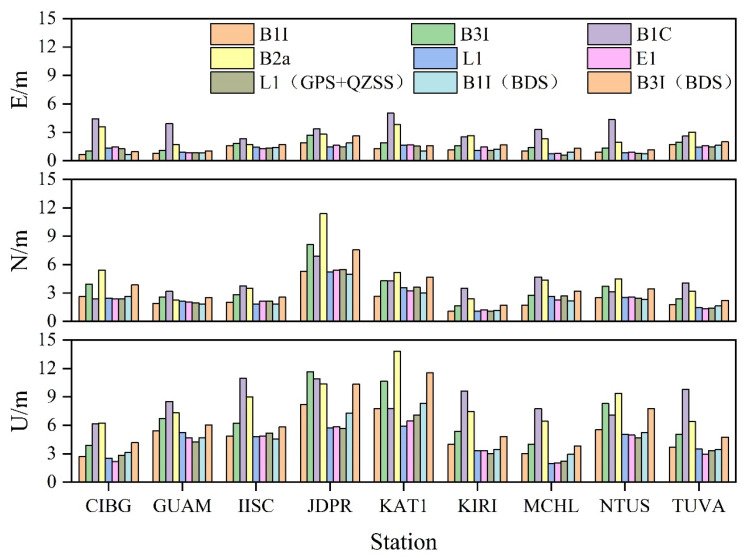
SPP positioning precision of Asia-Pacific stations.

Figure 4 shows the statistics of SPP precision for each frequency of the nine stations. The figure illustrates that the SPP of each system showed the best effect in the east direction (E direction), with the positioning precision ranging from 0.5 to 5 m. Moreover, the positioning error of most stations was around 1.5 m except for some frequencies, and only a few stations showed a slightly worse positioning effect, but still less than 3 m. However, the north direction (N direction) showed the second-best effect, with the positioning precision between 1 and 11 m, except for two stations, JDPR and KAT1, most of the frequency positioning errors of other stations were still below 3 m. The zenith direction (U direction) single frequency SPP effect was the worst, the positioning precision was between 1.5 and 13.5 m, and except for some frequencies, the positioning precision of other frequencies was about 6 m, and that of a few frequencies was about 3 m.

**Figure 5:**
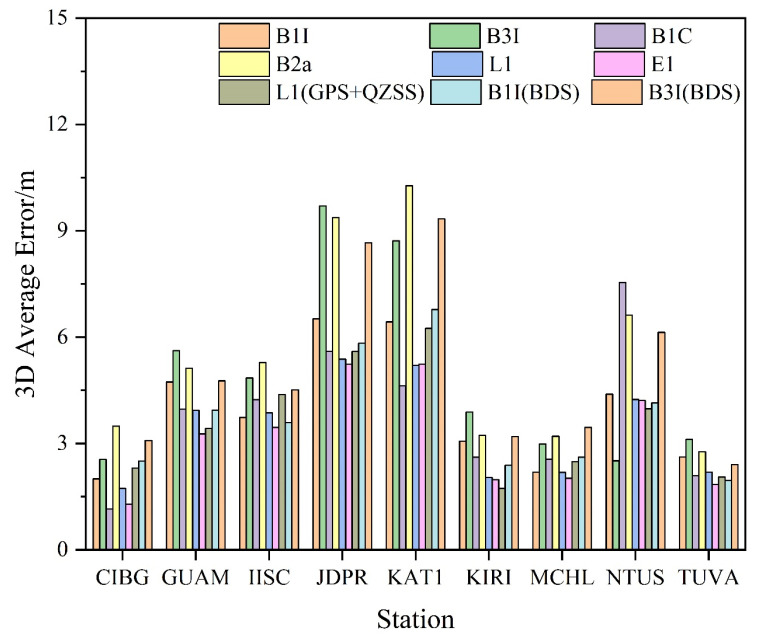
SPP 3D average positioning error of the station.

Figure 5 shows the average positioning deviation of each station in three-dimensional (3D) directions of different frequencies. Clearly, most of the stations were within 6 m of the 3D directional positioning deviation, and some of them can reach within 3 m, and only a few stations had slightly worse positioning precision for some frequencies. For BDS-3, the best 3D orientation deviation was B1C, which can reach 1.15 m; followed by B1I, which can reach 2 m. Further, B3I and B2a were slightly worse, which can reach 2.51 m and 2.77 m, respectively; while GPS and Galileo 3D orientation deviation can reach 1.73 m and 1.29 m, respectively. After adding BDS-2, no significant improvement was observed in the positioning deviation of B1I and B3I; after adding QZSS, a significant improvement was not observed even in the L1 positioning deviation. A combined analysis of Figure 4 indicates that due to the poor positioning precision in elevation direction, the positioning deviation in 3D direction was not very satisfactory. In a word, the positioning precision was superior in the following order: B1C > E1 > L1 > B1I > B3I > B2a.

### 3.2. PPP Convergence Analysis

**Figure 6:**
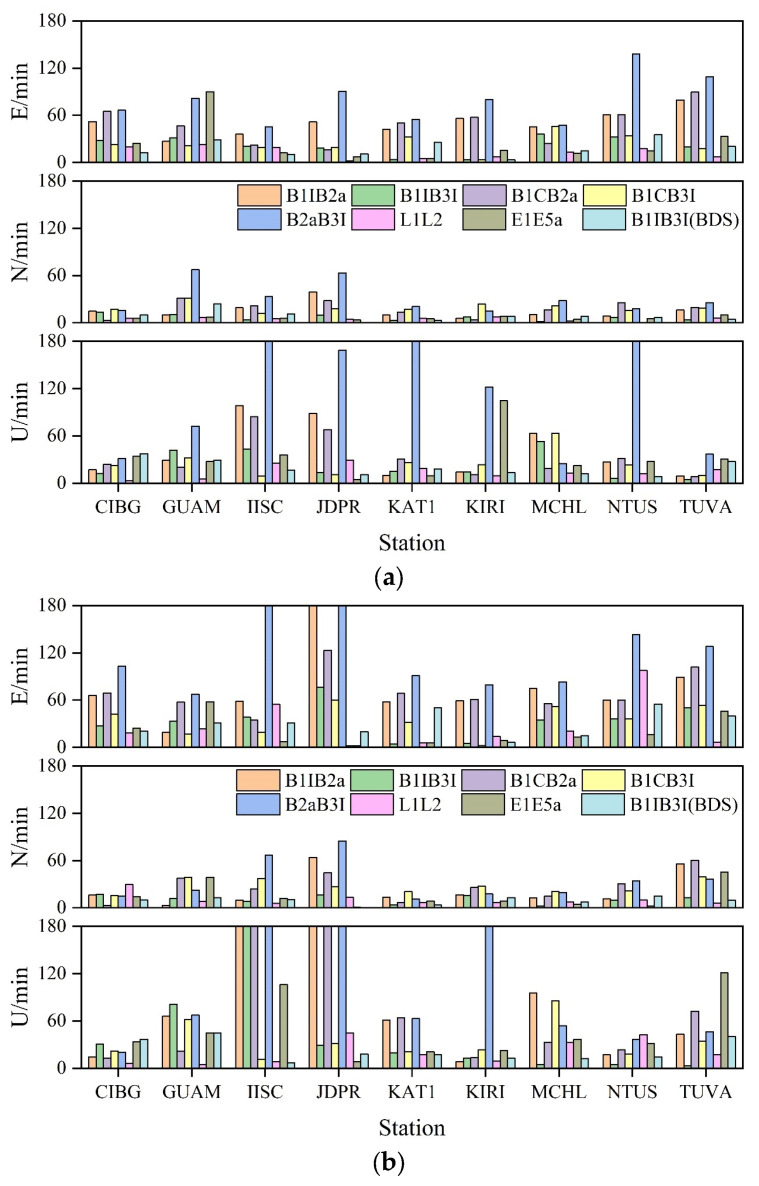
PPP convergence time of MGEX station. (**a**) Static PPP convergence time. (**b**) Kinematic PPP convergence time.

The convergence condition is defined as the error in the E, N, and U directions being less than 10 cm for 20 consecutive epochs. The convergence times of the static PPP and kinematic PPP for the nine selected stations in the Asia-Pacific region were counted, respectively, and the experimental results are shown in Figure 6. The results show that irrespective of the type of PPP; i.e., static or kinematic, the convergence time in N direction was better than that in the other two directions, which was probably due to the receiver type or the better spatial configuration of the satellite in the N direction than in the E direction.

The average convergence times of PPP for each frequency of the selected stations in static and kinematic modes are presented in Table 3. The results show that the convergence of L1L2 was the fastest, which was about 11 min in static mode and 19 min in kinematic mode; those of B1IB3I and E1E5a were slightly lower than the former, about 20 min in the static mode, and 28 min in the kinematic mode. Furthermore, B1CB3I showed slightly worse convergence, about 23 min in static mode and 32 min in kinematic mode; values for B1IB2a, B1CB2a were about 35 min in static mode and 57 min in kinematic mode; while B2aB3I showed the worst result, with convergence times of about 110 min in both static and kinematic modes. After the addition of BDS-2, the convergence time of B1IB3I in static mode was reduced to 15 min, and that in kinematic mode was reduced to 21 min, which were improved by 11% and 27%, respectively.

In a word, LIL2 showed the shortest convergence time, followed by B1IB3I and E1E5a, followed by B1CB3I, while B1CB2a and B1IB2a were slightly worse result and B2aB3 exhibited the worst value. However, with the addition of BDS-2, B1IB3I showed a significant improvement and was almost similar to L1L2.

### 3.3. PPP Precision Analysis

**Figure 7:**
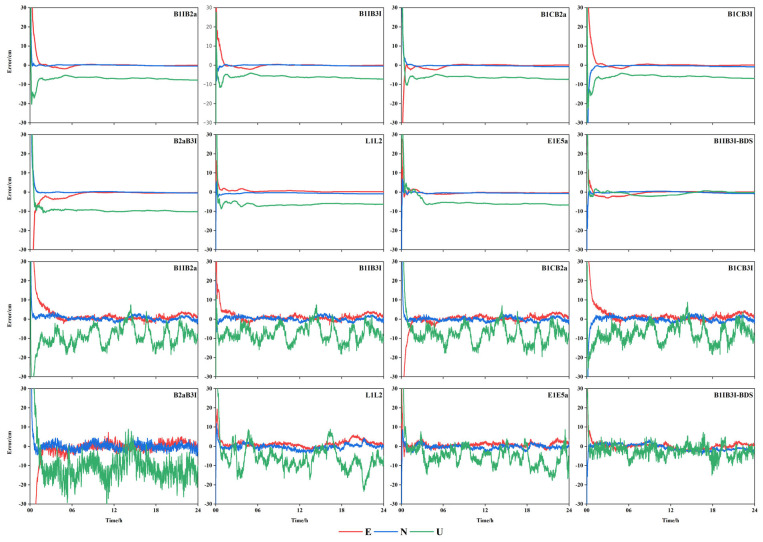
Time series of PPP for each frequency combination of MCHL stations.

The static PPP and kinematic PPP data of nine selected MGEX stations in the Asia-Pacific region were calculated, respectively. Considering the MCHL measurement station as an example, the time series graphs of each frequency combination in the E, N, and U directions were obtained, as shown in Figure 7.

Figure 7 shows that the difference in positioning precision of each frequency combination in static mode was not significant, but it was more obvious that B1IB2a, B1IB3I, B1CB3I, and B2aB3I converged slowly. After adding BDS-2, the convergence speed and positioning precision of B1IB3I improved significantly, in particular, in the elevation direction, while the positioning precision in the plane direction was comparable to those of GPS and Galileo, and the elevation direction was better than that of the latter two. In the kinematic mode, the positioning precision of all frequency combinations except B2aB3I was comparable; however, the convergence speed of B1IB2a, B1IB3I, B1CB3I, and B2aB3I was still worse, while the positioning precision of B2aB3I in the plane direction fluctuated more and the elevation direction was significantly worse than the other frequencies. After adding BDS-2, the convergence speed and positioning precision of B1IB3I improved significantly, irrespective of the plane direction or elevation direction, the positioning precision was significantly better than those of GPS and Galileo.

**Figure 8:**
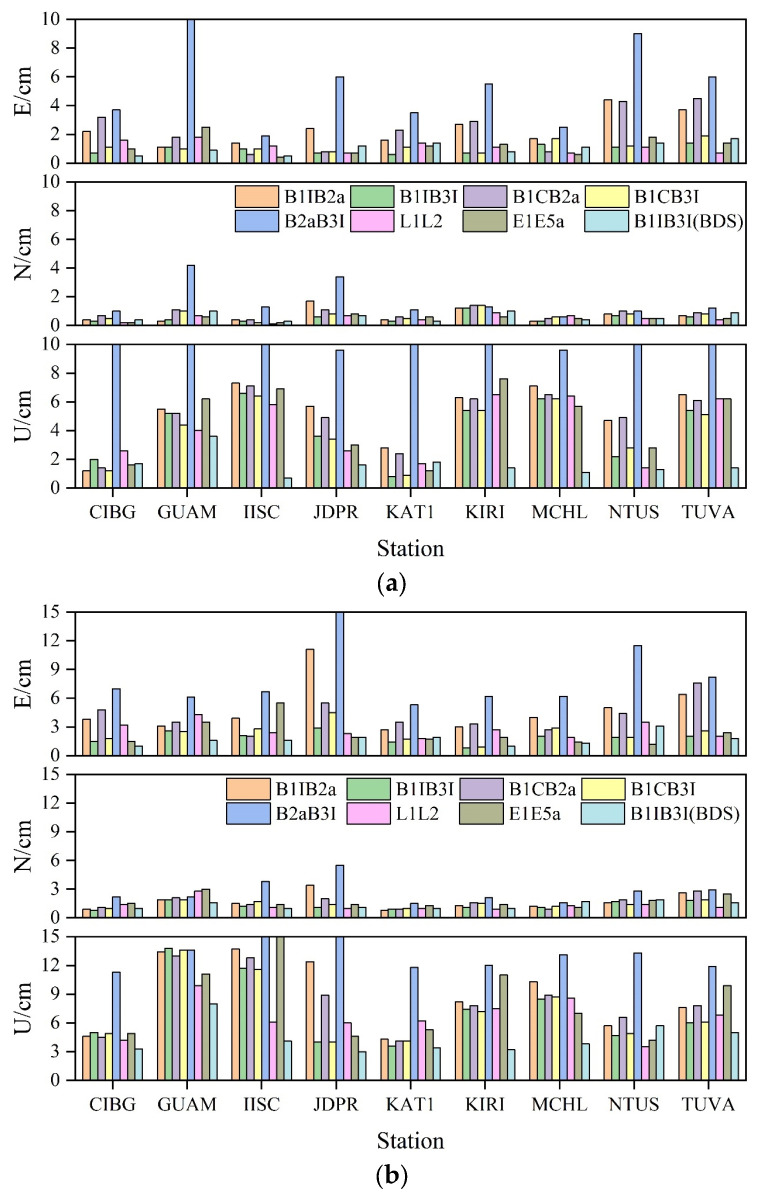
MGEX station PPP precision statistics. (**a**) Static PPP precision statistics. (**b**) Kinematic PPP precision statistics.

Figure 8 shows the positioning precision of PPP in E, N, and U directions for each frequency combination of nine stations. Similar to the convergence time mentioned above, and irrespective of the static or kinematic mode, the positioning precision in the N direction was still better than that in the E direction. Therefore, it was speculated that the reason was still that the space configuration of the satellite in the N direction was better than that in the E direction. A comprehensive analysis of the nine stations showed that, in static mode, B1IB3I, B1CB3I, L1L2, and E1E5a in the E direction exhibited the best positioning precision, B1IB2a and B1CB2a showed a slightly worse result, and B2aB3I exhibited the worst precision. In contrast, in the N direction, B1IB3I, L1L2, and E1E5a showed the best positioning precision, followed by B1IB2a and B1CB3I, B1CB2a showed a slightly worse result, and B2aB3I exhibited the worst positioning precision. In the U direction, B1CB3I, B1IB3I, and L1L2 showed the best positioning precision, followed by E1E5a, B1CB2a, and B1IB2a, and B2aB3I exhibited the worst positioning precision. After adding BDS-2, no significant change in B1IB3I plane direction was observed, and the elevation direction was improved by about 61%. In kinematic mode, B1B3 positioning precision was best in E direction, followed by B1CB3I, E1E5a, and L1L2; B1CB2a and B1B2a showed to be slightly worse, and B2aB3I precision was the worst. In the N direction, B1IB3I and L1L2 exhibited the best positioning precision, followed by B1CB3I, B1CB2a, B1IB2a, and E1E5a, which showed a slightly worse precision, and B2aB3I exhibited the worst positioning precision. In the U direction, L1L2, B1IB3I, and B1CB3I showed the best positioning precision, followed by B1CB2a and B1IB2a, followed by E1E5a, and B2aB3I showed the worst positioning precision. After adding BDS-2, B1IB3I increased by about 16% in E direction, no significant change occurred in N direction, and increased by about 38% in U direction.

**Figure 9:**
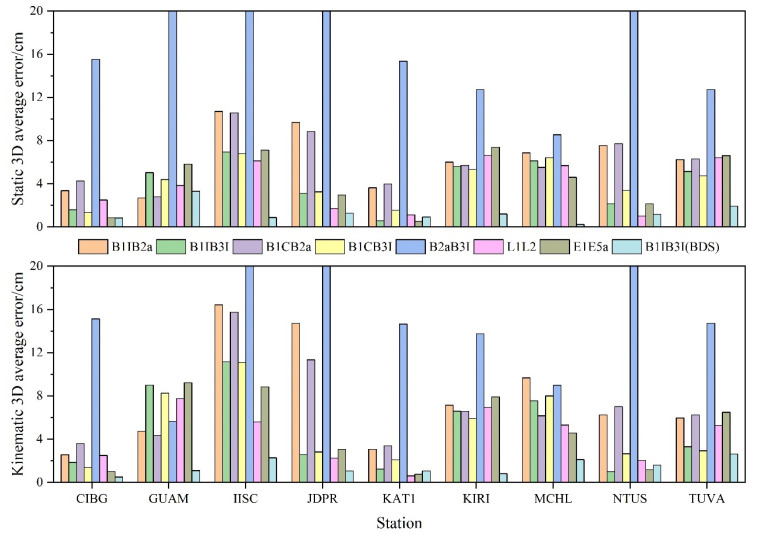
PPP 3D average positioning deviation.

The average positioning deviations of each station with different frequency PPP 3D in directions are presented in Figure 9. In static mode, the B1IB3I and B1CB3I positioning deviations were significantly better than L1L2 and E1E5a; those of B1IB2a and B1CB2a were slightly worse, and B2aB3I positioning deviation was significantly worse than the remaining frequency combinations. After adding BDS-2, B1IB3I positioning deviation was significantly improved, with an average improvement of about 67%, much more than those of L1L2 and E1E5a. In kinematic mode, the positioning deviation of B1IB3I and B1CB3I was similar to that of L1L2 and E1E5a, and that of some stations was better than the latter two. Positioning deviations of B1IB2a and B1CB2a were a little worse than the first four combinations at some stations. However, some stations showed a slightly larger gap, while B2aB3I was similar to the static results, and the gap was more obvious compared with other frequency combinations. After adding BDS-2, the results were consistent with the static results. The positioning deviation was significantly improved, about 70% on average, and its positioning deviation was much better than those of the L1L2 and E1E5a. A combined analysis of Figure 8 indicated that the positioning effect of each combination plane direction was better, and the N direction was significantly better than the E direction. However, the positioning precision in the elevation direction was obviously worse; thus, it may have a certain influence on the average deviation of 3D direction.

## 4. Conclusions

In order to verify the positioning performance of BDS-3 at each frequency in the Asia-Pacific region, in this study, observation data from nine MGEX stations and SPP and dual-frequency Ionosphere-free combinations PPP experiments were conducted for each frequency of BDS-3 by using the precision products provided by WHU. Furthermore, the results were compared with those of GPS and Galileo. Based on this, BDS-2 and QZSS were used to supplement BDS-3 and GPS B1IB3I/L1L2 SPP experiment and B1IB3I PPP experiment. Based on the results of this study, the following conclusions can be drawn:

(1) BDS-3 is better than GPS and Galileo in terms of both the number of satellites and the PDOP. The average number of visible satellites of BDS-3 is around 13, and the average PDOP can reach around 1.7. The average number of visible satellites of GPS is around 10 and the average PDOP can reach around 1.9. In contrast, the average number of visible satellites of Galileo is around 7 and the average PDOP is around 2.2. After adding BDS-2, the number of visible satellites of BDS can reach around 21, and the PDOP can reach around 1.3, which corresponds to a significant improvement.
(2) In terms of SPP precision, the positioning precision of each frequency in the E direction is the best, with most of the stations having a positioning precision of around 1.5 m. The positioning precision of each frequency in the N direction is mostly below 3 m, while that of each frequency in the U direction is around 6 m. After adding BDS-2 and QZSS, the positioning precision does not improve significantly. Overall, the positioning precision is in the following order: B1C > E1 > L1 > B1I > B3I > B2a.
(3) In terms of PPP convergence time, L1L2 shows the convergence time of about 11 min in static mode and 19 min in kinematic mode, while B1IB3I shows about 20 min in static mode and 28 min in kinematic mode, and the convergence time of other frequency combinations is slightly slower. After adding BDS-2, the convergence time of B1IB3I is reduced to 15 min in static mode and 21 min in kinematic mode, respectively, being improved by 11% and 27%, respectively. Overall, the convergence time is as follows: L1L2 > B1IB3I > E1E5a > B1CB3I > B1CB2a > B1IB2a > B2aB3I. After BDS-2 is added, the convergence times of B1IB3I and L1L2 are similar.
(4) In terms of PPP precision, the N direction is better than the E direction. In static mode, the positioning precision of the N direction is mostly within 2 cm, and that of only part of the frequency combination in the E direction can be within 2 cm. However, in kinematic mode, the positioning precision of the N direction is mostly within 3 cm, and most frequency combinations in the E direction are within 6 cm. In the 3D direction, most of the frequency combinations in the static mode are within 6 cm, and only some of them can reach 3 cm, while most of the frequency combinations in the kinematic mode are within 8 cm, and only a few can reach 4 cm. After adding BDS-2, the plane direction of B1IB3I in static mode is not significantly improved, the elevation direction is improved by about 61%, and the 3D direction is improved by about 67%. In kinematic mode, the E direction is improved by about 16%, the N direction does not change significantly, the U direction is improved by about 38%, and the 3D direction is improved by about 70%, which is significantly better than those of GPS and Galileo in terms of positioning precision. Overall, the order of positioning precision is as follows: B1IB3I > B1CB3I > L1L2 > E1E5a > B1B2a > B1CB2a > B2aB3I.

Although BDS-3 was in operation for more than three years, there are still few stations in the Asia-Pacific region that can accept the new BDS-3 frequency B1C/B2a; thus, only nine stations were selected for the experiment. Therefore, we are looking forward to more stations being added to the experiment as the receiver antennas at the MGEX ground sites are updated.

## Figures and Tables

**Table 1:** Basic information of MGEX station.

Station	Longitude	Latitude	Receiver Type	Antenna Type	Frequency
CIBG	106.849	−6.49	TRIMBLE ALLOY	LEIAR25.R4	B1I/B1C/B2a/B3I/L1/L2/E1/E5a
GUAM	144.868	13.589	SEPT POLARX5	JAVRINGANT_DM	B1I/B1C/B2a/B3I/L1/L2/E1/E5a
IISC	77.57	13.021	SEPT POLARX5	ASH701945E_M	B1I/B1C/B2a/B3I/L1/L2/E1/E5a
JDPR	73.024	26.207	TRIMBLE ALLOY	TWIVC6050	B1I/B1C/B2a/B3I/L1/L2/E1/E5a
KAT1	132.153	−14.376	SEPT POLARX5	LEIAR25.R3	B1I/B1C/B2a/B3I/L1/L2/E1/E5a
KIRI	172.923	1.355	SEPT POLARX5	TRM59800	B1I/B1C/B2a/B3I/L1/L2/E1/E5a
MCHL	148.145	−26.359	TRIMBLE ALLOY	TRM59800	B1I/B1C/B2a/B3I/L1/L2/E1/E5a
NTUS	103.68	1.346	LEICA GR50	LEIAR20	B1I/B1C/B2a/B3I/L1/L2/E1/E5a
TUVA	179.197	−8.525	SEPT POLARX5	JAVRINGANT_DM	B1I/B1C/B2a/B3I/L1/L2/E1/E5a

**Table 2:** Data processing strategies.

Options	Processing Strategies
Observation	Carrier phase and pseudo-range
Model	Ionosphere-free
Solution model	Single frequency pseudo-distance, static/kinematic
Observation interval	30 s
Frequency	BDS:B1I/B1C/B2a/B3I;QZSS:L1 GPS:L1/L2;Galileo:E1/E5a
satellite orbit and clock	WHU precision orbits and clock
Elevation cut off	10°
Parameter estimation method	Kalman filtering
Satellite phase center	igs20.atx
Cycle slip detection	GF + MW
Weighting scheme	Elevation dependent weight
Tropospheric delay	Saastamoinen
Tropospheric mapping function	VMF1
Ambiguity	Float
Receiver coordinates	Parameters estimation
Receiver clock error	Parameters estimation
Intersystem bias	WHU-BIA

**Table 3:** Average convergence time of PPP at MGEX station (min).

	B1IB2a	B1IB3I	B1CB2a	B1CB3I	B2aB3I	L1L2	E1E5a	B1IB3I(BDS)
Static	35	17	33	23	115	11	21	15
Kinematic	58	29	56	32	107	19	27	21

## Data Availability

All data can be available from Multi-GNSS Experiment (MGEX) or available from the corresponding author.
